# Effect of donor exclusion criteria on blood safety and volume of donations: a systematic review of modelling studies

**DOI:** 10.2471/BLT.25.294318

**Published:** 2026-01-22

**Authors:** Luce Mosselmans, Mart P Janssen, Hendrik B Feys, Emmy de Buck, Fritz Schiltz, Peter Vandenberghe, Philippe Vandekerckhove, Hans Van Remoortel

**Affiliations:** a Centre for Evidence-Based Practice, Belgian Red Cross-Flanders, Motstraat 42, 2800 Mechelen, Belgium.; b Transfusion Technology Assessment Department, Sanquin Research, Amsterdam, Kingdom of the Netherlands.; c Belgian Red Cross-Flanders, Blood Services, Mechelen, Belgium.; d Department of Haematology, University Hospitals Leuven, Leuven, Belgium.

## Abstract

**Objective:**

To assess the effect of easing bans and deferral policies on blood donation from men who have sex with men on blood safety and volume from modelling studies.

**Methods:**

We searched four databases (PubMed®, Embase®, CINAHL and Web of Science) for modelling studies on the impact of replacing bans or deferrals on blood donations from men who have sex with men with shorter deferral periods or no deferrals. We synthesized and compared findings from the different modelling studies, and assessed risk of bias and certainty of evidence.

**Findings:**

Fourteen publications were included in the study. All the studies estimated the human immunodeficiency virus (HIV) residual risk of an HIV-infected blood donation being received, going undetected through screening and entering the blood supply. Despite medium to large increases in risks (up to three-fold) in relaxing donor policies for men who have sex with men, the absolute HIV residual risk remained very low in all scenarios, ranging between 0.05 and 1.1 HIV-positive units per million donations. The increase in donors ranged from 0.04% to 2.10%. No models covered other transfusion-transmissible infections such as syphilis or hepatitis C.

**Conclusion:**

Modelled HIV residual risk estimates increased slightly with relaxed policies on donations from men who have sex with men. However, differences in risk and blood volume estimates between different policies are generally very small. To support decisions on easing donor policies for men who have sex with men, models should also quantify residual risks for non-HIV transfusion-transmissible infections, such as syphilis.

## Résumé

**Objectif:**

Évaluer l’effet de l’assouplissement des politiques d’interdiction et d’exclusion des hommes ayant des rapports sexuels avec des hommes sur la sécurité et le volume des dons de sang à partir d’études de modélisation.

**Méthodes:**

Nous avons effectué des recherches dans quatre bases de données (PubMed®, Embase®, CINAHL et Web of Science) afin de trouver des études de modélisation sur l’impact du remplacement des interdictions ou des reports de dons de sang des hommes ayant des rapports sexuels avec des hommes par des périodes de report plus courtes ou une absence de report. Nous avons synthétisé et comparé les résultats des différentes études de modélisation et évalué le risque de biais ainsi que la certitude des preuves.

**Résultats:**

Quatorze publications ont été reprises dans la présente étude. Toutes les études estimaient le risque résiduel qu’un don de sang infecté par le virus l’immunodéficience humaine (VIH) ne soit pas détecté lors du dépistage et intègre les réserves. Malgré des augmentations légères à moyennes des risques (jusqu’à 3 fois) liées à l’assouplissement des politiques de don pour les hommes ayant des rapports sexuels avec des hommes, le risque résiduel absolu lié au VIH est resté très faible dans tous les scénarios, oscillant entre 0,05 et 1,1 unité séropositive au VIH par million de dons. L’augmentation du nombre de donneurs variait entre 0,04 et 2,10%. Aucun modèle ne couvrait d’autres infections transmissibles par transfusion, telles que la syphilis ou l’hépatite C.

**Conclusion:**

Les estimations modélisées du risque résiduel lié au VIH ont légèrement augmenté avec l’assouplissement des politiques relatives aux dons des hommes ayant des rapports sexuels avec des hommes. Cependant, les différences entre les estimations du risque et du volume sanguin selon les différentes politiques sont généralement très faibles. Afin d’étayer les décisions relatives à l’assouplissement des politiques de don de sang pour les hommes ayant des rapports sexuels avec des hommes, les modèles devraient également quantifier les risques résiduels pour les infections transmissibles par transfusion autres que le VIH, telles que la syphilis.** **

## Resumen

**Objetivo:**

Evaluar, a partir de estudios de modelización, el efecto de flexibilizar las prohibiciones y las políticas de aplazamiento de la donación de sangre por parte de hombres que tienen relaciones sexuales con hombres en la seguridad de la sangre y el volumen de donaciones.

**Métodos:**

Se realizaron búsquedas en cuatro bases de datos (PubMed®, Embase®, CINAHL y Web of Science) de estudios de modelización sobre el impacto de sustituir las prohibiciones o los aplazamientos de la donación de sangre por parte de hombres que tienen relaciones sexuales con hombres por periodos de aplazamiento más cortos o por la ausencia de aplazamiento. Se sintetizaron y compararon los resultados de los distintos estudios de modelización y se evaluaron el riesgo de sesgo y la certeza de la evidencia.

**Resultados:**

Se incluyeron catorce publicaciones en el estudio. Todos los estudios estimaron el riesgo residual del virus de la inmunodeficiencia humana (VIH) de que una donación de sangre infectada por VIH fuera aceptada, no detectada durante el cribado y entrara en el suministro de sangre. A pesar de aumentos moderados a grandes en los riesgos (hasta tres veces) al flexibilizar las políticas de donación para hombres que tienen relaciones sexuales con hombres, el riesgo residual absoluto de VIH se mantuvo muy bajo en todos los escenarios, oscilando entre 0,05 y 1,1 unidades positivas para VIH por millón de donaciones. El aumento del número de donantes osciló entre el 0,04% y el 2,10%. Ningún modelo abordó otras infecciones transmisibles por transfusión, como la sífilis o la hepatitis C.

**Conclusión:**

Las estimaciones modelizadas del riesgo residual de VIH aumentaron ligeramente con la flexibilización de las políticas de donación por parte de hombres que tienen relaciones sexuales con hombres. No obstante, las diferencias en las estimaciones de riesgo y de volumen de sangre entre las distintas políticas son, por lo general, muy pequeñas. Para respaldar las decisiones sobre la flexibilización de las políticas de donación por parte de hombres que tienen relaciones sexuales con hombres, los modelos también deberían cuantificar los riesgos residuales de infecciones transmisibles por transfusión distintas del VIH, como la sífilis.

## ملخص

**الغرض:**

تقييم تخفيف عمليات الحظر وسياسات التأجيل المفروضة على التبرع بالدم من الرجال الذين يمارسون الجنس مع الرجال، وتقييم أثر ذلك على سلامة الدم وكميته، وذلك استنادًا إلى دراسات وضع النماذج.

**الطريقة:**

بحثنا في أربع قواعد بيانات (®PubMed، و®Embase، وCINAHL، وWeb of Science) عن دراسات وضع النماذج لتأثير استبدال عمليات الحظر أو التأجيلات المفروضة على التبرع بالدم من الرجال الذين يمارسون الجنس مع الرجال، بفترات تأجيل أقصر أو إلغاء التأجيلات. قمنا بتجميع ومقارنة نتائج الدراسات المختلفة لوضع النماذج، وتقييم مخاطر التحيز وموثوقية الأدلة.

**النتائج:**

شملت الدراسة أربعة عشر منشورًا. قدّرت جميع الدراسات خطر الإصابة بفيروس نقص المناعة البشرية (HIV) المتبقي في حال استلام تبرع دم مصاب بالفيروس دون اكتشافه خلال الفحص ودخوله إلى مخزون الدم. على الرغم من الزيادات المتوسطة إلى الكبيرة في المخاطر (حتى ثلاثة أضعاف) عند تخفيف سياسات التبرع بالدم للرجال الذين يمارسون الجنس مع رجال، إلا إن خطر الإصابة المطلق بفيروس نقص المناعة البشرية المتبقي ظل منخفضًا جدًا في جميع السيناريوهات، حيث تراوح بين 0.05 و1.1 وحدة دم مصابة بالفيروس لكل مليون تبرع. وتراوحت الزيادة في عدد المتبرعين بين %0.04 و%2.10. ولم تغط أي من النماذج أمراضًا أخرى تنتقل عن طريق نقل الدم، مثل الزهري أو التهاب الكبد الوبائي سي.

**الاستنتاج:**

ارتفعت تقديرات خطر الإصابة بفيروس نقص المناعة البشرية المتبقي، وفقًا للنماذج، بشكل طفيف مع تخفيف سياسات التبرع بالدم من الرجال الذين يمارسون الجنس مع رجال. ومع ذلك، فإن الاختلافات في تقديرات المخاطر وكمية الدم بين السياسات المختلفة ضئيلة جدًا بشكل عام. ولدعم القرارات المتعلقة بتخفيف سياسات التبرع بالدم على الرجال الذين يمارسون الجنس مع رجال، ينبغي أن تُحدد النماذج أيضًا كميًا المخاطر المتبقية للأمراض الأخرى التي تنتقل عن طريق نقل الدم، مثل الزهري.

## 摘要

**目的:**

旨在基于模型研究评估放宽针对男男性行为者的献血禁令并制定允许其延缓献血的政策对血液安全和献血量的影响。

**方法:**

我们搜索了四个数据库【PubMed￼^®^、Embase￼^®^、护理和联合卫生文献累积索引 (CINAHL) 和 Web of Science】以查找涉及以下内容的模型研究：将禁止或延缓男男性行为者献血的政策调整为缩短或取消其献血延缓期所造成的影响。我们综合考虑和对比了不同模型研究的结果，并评估了偏倚风险和证据的确定性。

**结果:**

共有十四份出版物被纳入了本项研究中。所有模型研究都预估了因接受人类免疫缺陷病毒（艾滋病毒）感染者献血、在筛选过程中未能检测出病毒并最终让血液进入血液供应系统而导致的艾滋病毒残余风险。虽然放宽针对男男性行为者的献血者政策导致残余风险适度或大幅上升（最高可达三倍），但是在所有情景下，艾滋病毒的绝对残余风险始终很低，每一百万份血液中仅有 0.05 至 1.1 份的艾滋病毒检测结果呈阳性。献血者人数却增加了 0.04% 至 2.10%。所有模型研究均未涵盖其他经输血传播的感染，例如梅毒或丙型肝炎。

**结论:**

模型研究结果显示，放宽针对男男性行为者的献血政策预计将会导致艾滋病毒残余风险小幅度上升。但是通常这两种不同政策下产生的艾滋病毒残余风险和献血量差异预计都会非常小。如要支持制定有关放宽针对男男性行为者的献血者政策的决策，这类模型研究还应量化除艾滋病毒以外其他经输血传播的感染（例如梅毒）的残余风险。

## Резюме

**Цель:**

С помощью исследований моделирования оценить воздействие ослабления прямых запретов и политик отвода от донорства для мужчин, практикующих секс с мужчинами, на объем донорской крови и ее безопасность.

**Методы:**

Авторы провели поиск по четырем базам данных (PubMed®, Embase®, CINAHL и Web of Science) на предмет исследований моделирования. В рамках этих исследований оценивалось воздействие перехода от прямых запретов на донорство (или отвода от него) для мужчин, практикующих секс с мужчинами, к более коротким срокам отвода (или их полной отмене). Авторы объединили и сравнили результаты различных исследований моделирования, а также оценили риск систематической ошибки и степень достоверности полученных данных.

**Результаты:**

В исследование были включены четырнадцать публикаций. Во всех исследованиях оценивался остаточный риск передачи вируса иммунодефицита человека (ВИЧ) в результате переливания ВИЧ-инфицированной донорской крови при условии, что инфицированная кровь прошла незамеченной через системы скрининга и попала в кровоток реципиента. Несмотря на увеличение риска (от умеренного до значительного, вплоть до трехкратного) в условиях ослабленных политик донорства для мужчин, практикующих секс с мужчинами, абсолютный остаточный риск инфицирования ВИЧ оставался очень низким во всех сценариях, составляя от 0,05 до 1,1 ВИЧ-положительной дозы на миллион случаев сдачи крови. Рост количества доноров составил от 0,04 до 2,10%. Ни в одной из моделей не рассматривался риск для других инфекций, передающихся при переливании крови, таких как сифилис или гепатит С.

**Вывод:**

При ослаблении политик отвода от донорства для мужчин, практикующих секс с мужчинами, моделированный риск передачи ВИЧ незначительно возрастает. Однако различия в оценке риска и объема сдаваемой крови между различными политиками оказываются, как правило, очень незначительными. Для обоснования решений о смягчении донорских политик в отношении мужчин, практикующих секс с мужчинами, модели также должны количественно оценивать остаточные риски трансфузионной передачи инфекций, отличных от ВИЧ, таких как сифилис.

## Introduction

The deferral, that is, the temporary or permanent exclusion, of blood donors at higher risk of having transfusion-transmissible infections – such as men who have sex with men, commercial sex workers or injection drug users – is a strategy to increase the safety of blood products.^1^^,^^2^ This strategy, along with pathogen screening, was introduced as a response to transfusion-transmitted human immunodeficiency virus (HIV) infection in the initial years of the acquired immunodeficiency disease syndrome (AIDS) epidemic. However, delays in adopting adequate risk reduction strategies in the 1970s to 1990s, even after the causative agent of AIDS was identified, resulted in thousands of preventable infections and related disease worldwide^3^. Subsequent legal inquiries in several countries, including France, Japan and the United Kingdom of Great Britain and Northern Ireland, criticized policy-makers and blood service authorities for their inaction and failures in preserving blood safety.^3^^,^^4^ These historical failures underscored the need for precautionary, evidence-based approaches to donor selection and testing, and they shape the ethical and operational frameworks governing blood safety today.^5^ Deferrals for men who have sex with men are increasingly seen as stigmatizing, especially in high-income countries with relatively low HIV prevalence and current sensitive screening tests.^6^^,^^7^ Nonetheless, in Europe, the incidence of HIV is disproportionately high in some subpopulations including men who have sex with men. According to the European Centre for Disease Prevention and Control, nearly half of cases of HIV infection in western Europe in 2022 were men who have sex with men.^8^

In the past decade, blood establishments in several countries (Australia, Canada, France, Germany, Kingdom of the Netherlands, United Kingdom and United States of America) have modified blood donor criteria by shortening the length of deferral for men who have sex with men, or by replacing exclusion with a risk assessment for all blood donors, regardless of sex or sexual orientation (also referred to as individual risk assessment).^9^^,^^10^ In 2022, we conducted a systematic review of the risk of transfusion-transmitted infections in blood donors and deferral policies for men who have sex with men, and concluded that observational studies yielded limited evidence of the impact of 3-month deferral for men who have sex with men or risk-based deferrals on the  risk of transfusion-transmissible infections.^11^ However, our review did not include evidence from modelling studies. Modelling studies may offer additional information to support decision-making on donor deferral policies.

In this article, we therefore aim to identify, critically appraise and synthesize modelling studies that estimated the impact of easing deferral policies for men who have sex with men on the residual risk of transfusion-transmissible infections and/or the number of donations. This review is intended to support blood establishments in (re)evaluating deferral policies for men who have sex with men through modelled data from different countries, and summarizing the methods available to assess the risk of  transfusion-transmissible infections within their specific settings. We do not aim to make any policy recommendations, as each jurisdiction should interpret the risks within their local context.

## Methods

This systematic review was registered in PROSPERO (CRD42024524737) and followed the Preferred Reporting Items for Systematic Reviews and Meta-Analysis (PRISMA) guidelines^12^ (online repository).^13^

### Eligibility criteria

Studies addressing the following PICOD question were included, “In donors (population), does easing the deferral policy for donors who are men who have sex with men (intervention versus comparator) have an impact on the blood safety or volume (outcomes) in modelling studies (design)?” We reviewed studies based on predefined criteria (online repository).^13^

### Data sources and searches

We searched four databases on 27 April 2025: PubMed®, Embase®, CINAHL and Web of Science. We developed search strings according to the eligibility criteria and adapted to each database (Box 1).

Box 1 **Search strategy, by database for studies on the effect of donor exclusion criteria on blood safety and volume of donations**

***PubMed®***

(“blood donors”[Mesh] OR “blood donor”[TIAB] OR “blood donors”[TIAB] OR “blood donation”[Mesh] OR “blood donation”[TIAB] OR “blood donations”[TIAB] OR “blood banks”[Mesh] OR “blood banks”[TIAB] OR “blood bank”[TIAB] OR “blood service”[TIAB] OR “blood services”[TIAB] OR “blood center”[TIAB] OR “blood centers”[TIAB] OR “blood centre”[TIAB] OR “blood centres”[TIAB] OR “blood transfusion”[Mesh] OR “transfusion”[TIAB])

AND

(“hiv infections”[Mesh] OR “HIV”[TIAB] OR “hepatitis b virus”[Mesh] OR “hepatitis b”[TIAB] OR “HBV”[TIAB] or “hepatitis c”[TIAB] OR “HCV”[TIAB] OR “treponemal infections”[Mesh] OR “syphilis”[TIAB] OR “treponema pallidum”[TIAB] OR “emerging”[TIAB] OR “residual risk”[TIAB] or “infection”[TIAB] OR “infections”[TIAB] OR “transfusion-transmitted”[TIAB] OR “transfusion transmissible”[TIAB] OR “TTI”[TIAB] OR “blood safety”[Mesh] OR “blood safety”[TIAB] or “transfusion safety”[TIAB])

AND

(“models, theoretical”[Mesh] OR “models,statistical”[Mesh] OR “bayes theorem”[Mesh] OR “model”[TIAB] OR “models”[TIAB] OR “modelling”[TIAB] OR “modelling”[TIAB] OR “modelled”[TIAB] or “modelled”[TIAB] OR “computer simulation”[Mesh] OR” simulation”[TIAB] OR “predict”[TIAB] OR “prediction”[TIAB] OR “predicted”[TIAB] OR “estimate”[TIAB] OR “estimates”[TIAB] OR “estimated”[TIAB] OR “estimation”[TIAB] OR “scenario”[TIAB] OR “scenarios”[TIAB])

AND

(“deferral”[TIAB] OR “ban”[TIAB] OR “lifetime”[TIAB] OR “risk assessment”[TIAB] OR “men who have sex with men”[TIAB] OR “MSM”[TIAB] OR “men who have had sex with men”[TIAB] OR ”homosexuality, male”[Mesh], OR “gay”[TIAB] OR “bisexual”[TIAB] OR “sexual”[TIAB] OR “donor selection”[Mesh] OR “selection”[TIAB])

***Embase®***

(‘blood donors’/exp OR ‘blood donor’:ti,ab OR ‘blood donors’:ti,ab OR ‘blood donation’/exp OR ‘blood donation’:ti,ab OR ‘blood donations’:ti,ab OR ‘blood banks’/exp OR ‘blood banks’:ti,ab OR ‘blood bank’:ti,ab OR ‘blood service’:ti,ab OR ‘blood services’:ti,ab OR ‘blood centre’:ti,ab OR ‘blood centres’:ti,ab OR ‘blood centre’:ti,ab OR ‘blood centres’:ti,ab OR ‘blood transfusion’/exp OR transfusion:ti,ab)

AND

(‘hiv infections’/exp OR HIV:ti,ab OR ‘hepatitis b virus’/exp OR ‘hepatitis b’:ti,ab OR HBV:ti,ab OR ‘hepatitis c’:ti,ab OR HCV:ti,ab OR ‘treponemal infections’/exp OR syphilis:ti,ab OR ‘*Treponema pallidum*’:ti,ab OR ‘communicable disease, emerging’/exp OR emerging:ti,ab OR ‘residual risk’:ti,ab OR‘infection:ti,ab OR infection’:ti,ab OR ‘transfusion-transmitted’:ti,ab OR ‘transfusion transmissible’:ti,ab OR TT’:ti,ab OR ‘blood safety’/exp OR ‘blood safety’:ti,ab OR ‘transfusion safety’:ti,ab)

AND

(‘models, theoretical’/exp OR ‘models, statistical’/exp OR ‘bayes theorem’/exp OR model:ti,ab OR models:ti,ab OR modelling:ti,ab OR modeling:ti,ab OR modelled:ti,ab OR modelled:ti,ab OR ‘computer simulation’/exp OR simulation:ti,ab OR predict:ti,ab OR prediction:ti,ab OR predicted:ti,ab OR estimate:ti,ab OR estimates:ti,ab OR estimated:ti,ab OR estimation:ti,ab OR scenario:ti,ab OR scenarios:ti,ab)

AND

(deferral:ti,ab OR ban:ti,ab OR lifetime:ti,ab OR ‘risk assessment’:ti,ab OR ‘men who have sex with men’:ti,ab OR MSM:ti,ab OR ‘men who have had sex with men’:ti,ab OR homosexuality, male’/exp OR gay:ti,ab OR bisexual:ti,ab OR ‘donor selection’/exp OR selection:ti,ab)

***CINAHL***

((MH “blood donors+”) OR (TI “blood donor” OR AB “blood donor”) OR (TI “blood donors” OR AB “blood donors”) OR (MH “blood donation+”) OR (TI “blood donation” OR AB “blood donation”) OR (TI “blood donations” OR AB “blood donations”) OR (MH “blood banks+”) OR (TI “blood banks” OR AB “blood banks”) OR (TI “blood bank” OR AB “blood bank”) OR (TI “blood service” OR AB “blood service”) OR (TI “blood services” OR AB “blood services”) OR (TI “blood center” OR AB “blood center”) OR (TI “blood centers” OR AB “blood centers”) OR (TI “blood centre” OR AB “blood centre”) OR (TI “blood centres” OR AB “blood centres”) OR (MH “blood transfusion+”) OR (TI transfusion OR AB transfusion))

AND

((MH “hiv infections+”) OR (TI HIV OR AB HIV) OR (MH “hepatitis b virus+”) OR (TI “hepatitis b” OR AB “hepatitis b”) OR (TI HBV OR AB HBV) OR (TI “hepatitis c” OR AB “hepatitis c”) OR (TI HCV OR AB HCV) OR (MH “treponemal infections+”) OR (TI syphilis OR AB syphilis) OR (TI “treponema pallidum” OR AB “treponema pallidum”) OR (MH “communicable disease, emerging+”) OR (TI emerging OR AB emerging) OR (TI “residual risk” OR AB “residual risk”) OR (TI infection OR AB infection) OR (TI infections OR AB infections) OR (TI transfusion-transmitted” OR AB transfusion-transmitted”) OR (TI “transfusion transmissible” OR AB “transfusion transmissible”) OR (TI TTI OR AB TTI) OR (MH “blood safety+”) OR (TI “blood safety” OR AB “blood safety”) OR (TI “transfusion safety” OR AB “transfusion safety”))

AND

((MH “models, theoretical+”) OR (MH “models, statistical+”) OR (MH “bayes theorem+”) OR (TI model OR AB model) OR (TI models OR AB models) OR (TI modelling OR AB modelling) OR (TI modeling OR AB modeling) OR (TI modelled OR AB modelled) OR (TI modelled OR AB modelled) OR (MH “computer simulation+”) OR (TI simulation OR AB simulation) OR (TI predict OR AB predict) OR (TI prediction OR AB prediction) OR (TI predicted OR AB predicted) OR (TI estimate OR AB estimate) OR (TI estimates OR AB estimates) OR (TI estimated OR AB estimated) OR (TI estimation OR AB estimation) OR (TI scenario OR AB scenario) OR (TI scenarios OR AB scenarios))

AND

((TI deferral OR AB deferral) OR (TI ban OR AB ban) OR (TI lifetime OR AB lifetime) OR (TI “risk assessment” OR AB “risk assessment”) OR (TI “men who have sex with men” OR AB “men who have sex with men”) OR (TI men who have sex with men OR AB men who have sex with men) OR (TI “men who have had sex with men” OR AB “men who have had sex with men”) OR (MH “homosexuality, male+”) OR (TI gay OR AB gay) OR (TI bisexual OR AB bisexual) OR (MH “donor selection+”) OR (TI selection OR AB selection))

***Web of Science***

(ALL = “blood donors” OR (TI = “blood donor” OR AB = “blood donor”) OR (TI = “blood donors” OR AB = “blood donors”) OR ALL = ”blood donation” OR (TI = ”blood donation” OR AB = ”blood donation”) OR (TI = ”blood donations” OR AB = ”blood donations”) OR ALL = ”blood banks” OR (TI = ”blood banks” OR AB = ”blood banks”) OR (TI = ”blood bank” OR AB = ”blood bank”) OR (TI = ”blood service” OR AB = ”blood service”) OR (TI = ”blood services” OR AB = ”blood services”) OR (TI = ”blood centre” OR AB = ”blood centre”) OR (TI = ”blood centres” OR AB = ”blood centres”) OR (TI = ”blood centre” OR AB = ”blood centre”) OR (TI = ”blood centres” OR AB = ”blood centres”) OR ALL = ”blood transfusion” OR (TI = transfusion OR AB = transfusion))

AND

(ALL = “hiv infections” OR (TI = HIV OR AB = HIV) OR ALL = ”hepatitis b virus” OR (TI =  “hepatitis b” OR AB = “hepatitis b”) OR (TI = HBV OR AB = HBV) OR (TI =  “hepatitis c” OR AB = “hepatitis c”) OR (TI = HCV OR AB = HCV) OR ALL = “treponemal infections” OR (TI = syphilis OR AB = syphilis) OR (TI = “*Treponema pallidum*” OR AB = “*Treponema pallidum*”) OR ALL = ”communicable disease, emerging” OR (TI = emerging OR AB = emerging) OR (TI = “residual risk” OR AB = “residual risk”) OR (TI = infection OR AB = infection) OR (TI = infections OR AB = infections) OR (TI = transfusion-transmitted OR AB = transfusion-transmitted) OR (TI = ”transfusion transmissible” OR AB = “transfusion transmissible”) OR (TI = TTI OR AB = TTI) OR ALL = “blood safety” OR (TI = “blood safety” OR AB = “blood safety”) OR (TI = “transfusion safety” OR AB = “transfusion safety”))

AND

(ALL = “models, theoretical” OR ALL = “models, statistical” OR ALL = “bayes theorem” OR (TI = model OR AB = model) OR (TI = models OR AB = models) OR (TI = modelling OR AB = modelling) OR (TI = modeling OR AB = modeling) OR (TI = modelled OR AB = modelled) OR (TI = modelled OR AB = modelled) OR ALL = “computer simulation” OR (TI = simulation OR AB = simulation) OR (TI = predict OR AB = predict) OR (TI = prediction OR AB = prediction) OR (TI = predicted OR AB = predicted) OR (TI = estimate OR AB = estimate) OR (TI = estimates OR AB = estimates) OR (TI = estimated OR AB = estimated) OR (TI = estimation OR AB = estimation) OR (TI = scenario OR AB = scenario) OR (TI = scenarios OR AB = scenarios))

AND

((TI = deferral OR AB = deferral) OR (TI = ban OR AB = ban) OR (TI = lifetime OR AB = lifetime) OR (TI = “risk assessment” OR AB = “risk assessment”) OR (TI = “men who have sex with men” OR AB = ”men who have sex with men”) OR (TI = MSM OR AB = MSM) OR (TI = “men who have had sex with men” OR AB = “men who have had sex with men”) OR ALL = “Homosexuality, Male” OR (TI = gay OR AB = gay) OR (TI = bisexual OR AB = bisexual) OR (TI = sexual OR AB = sexual) OR ALL = “donor selection” OR (TI = selection OR AB = selection))

### Study selection

We screened articles based on title, abstract and full text according to the eligibility criteria. We screened the reference lists of the included peer-reviewed journal articles and the first 20 similar articles displayed in PubMed® for additional relevant records.

### Data extraction and synthesis

We extracted the following data: type of model; setting; the modelled intervention (transfusion-transmissible infection(s) assessed and deferral policy for men who have sex with men at baseline and modelled); modelled outcomes (blood safety and number of donations); and the modelling assumptions made.

We synthesized and compared findings from the different modelling studies and used a narrative synthesis approach to assess the results based on length of deferral. No meta-analysis was conducted. 

### Risk of bias and grading 

We assessed the risk of bias using a grading scale^14^ that was developed based on the modelling good practices checklists.^15^^,^^16^ The grading scale has 14 questions on definitions, model methods, model inputs, fitting and validation, results, and conflicts of interest. Each question was scored 0, 1 or 2 points, resulting in an overall score ranging from 0 to 28. We categorized risk of bias as follows: ≤ 14 as high risk of bias; 15–18 as medium risk of bias; and ≥ 19 as low risk of bias. We used the Grading of Recommendations, Assessment, Development and Evaluation (GRADE) approach to assess the overall certainty of the modelled evidence for each outcome and sorted by baseline versus modelled policy on men who have sex with men.^17^ We initially graded outcomes from modelling studies as high certainty evidence and then downgraded as required based on risk of bias, indirectness, imprecision, inconsistency and risk of publication bias.

Two authors undertook the study selection, data extraction and risk of bias assessment independently and they resolved discrepancies by discussion. Where necessary, they consulted a third reviewer.

## Results

### Study selection and features

**Fig. 1:**
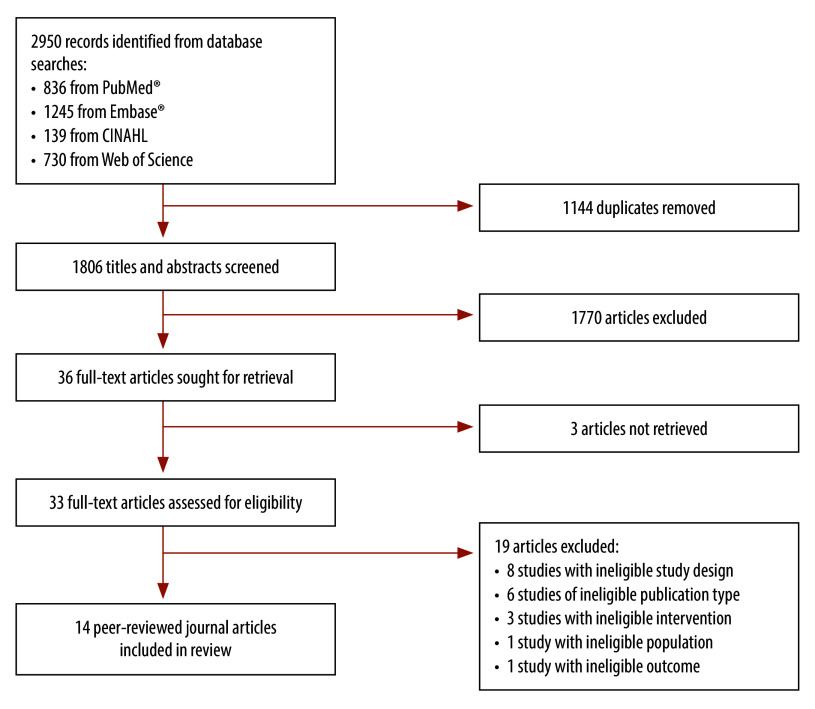
Flowchart of the selection of modelling studies on the effect of donor exclusion criteria on blood safety and volume of donations

We identified 2950 records through our search. After removing duplicates and screening titles and abstracts, we assessed 33 full-text articles, of which 14 met the inclusion criteria (Fig. 1 and online repository).^13^ Table 1 shows the characteristics of the 14 included studies. One study modelled the complete removal of risk-based deferrals, including for men who have sex with men, injection drug users and commercial sex workers.^26^ Five models were deterministic mathematical equation models (i.e. parameters are fixed),^19^^,^^21^^–^^23^^,^^25^ while the other nine used stochastic models (i.e. parameters are described by probability distributions) where the final outcome was determined by Monte Carlo simulations.^18^^,^^20^^,^^24^^,^^26^^–^^31^ All 14 studies modelled the risk of HIV transmission; one study also included the risk of hepatitis B virus (HBV) transmission.^20^ We did not find any studies investigating the risk of hepatitis C virus (HCV) or syphilis transmission. One study assessed the risk of infections in plasma donations,^30^ while the other studies estimated risks in blood donations.

**Table 1:** Characteristics of the included studies on the effect of donor exclusion criteria on blood safety and volume of donations

First author	Country	Modelling method (model type)^a^	Deferral policy on men who have sex with men^b^		Outcomes modelled	
			Baseline	Modelled	Blood safety	Volume
Germain et al., 2003^18^	USA and Canada	Monte Carlo simulation (stochastic)	Lifetime	1 year	HIV residual risk per million donations	Additional number of new donors
Soldan et al., 2003^19^	United Kingdom	Mathematical model (deterministic)	Lifetime	1 year	Additional number of HIV-positive blood units released	Additional number of new donors
Anderson et al., 2009^20^	USA	Monte Carlo simulation (stochastic)	Lifetime	5 years; 1 year	Additional number of HIV-positive or hepatitis B virus-positive blood units released	Additional number of new donors
Davison et al., 2011^21^	United Kingdom	Mathematical equation model (deterministic)	Lifetime	5 years	HIV residual risk per million donations	Not reported
Pillonel et al., 2012^22^	France	Mathematical equation model (deterministic)	Lifetime	IRA	HIV residual risk per million donations	Additional donor-years
Davison et al., 2013^23^	United Kingdom	Mathematical equation model (deterministic)	Lifetime	1 year	HIV residual risk per million donations	Additional number of new donors
Germain et al., 2014^24^	USA and Canada	Monte Carlo simulation (stochastic)	Lifetime	5 years	Number of additional HIV-positive blood units released	Additional number of new donors
Ginsberg et al., 2016^25^	Israel	Mathematical equation model (deterministic)	Lifetime	5 years; 1 year; IRA	HIV cases transmitted in the next decade	Additional number of new donations
Yang et al., 2016^26^	USA	Monte Carlo simulation (stochastic)	Lifetime	No risk deferral	Additional number of HIV-positive blood units released	Additional number of new donations
Davison et al., 2019^27^	United Kingdom and Canada	Monte Carlo simulation (stochastic)	Lifetime	1 year	HIV residual risk per million donations	Not reported
			5 years	1 year		
O’Brien et al., 2020^28^	Canada	Monte Carlo simulation (stochastic)	1 year	3 months	HIV residual risk per million donations	Additional number of new donors
Pillonel et al., 2020^29^	France	Monte Carlo simulation (stochastic)	1 year	4 months; IRA	HIV residual risk per million donations	Additional number of new donors
Aubé et al., 2022^30^	Canada	Bayesian network with Monte Carlo simulation (stochastic)	3 months for plasma donors	IRA for plasma donations	Probability of obtaining a plasma pool with an HIV viral load	Not reported
Caffrey et al., 2022^31^	Canada	Monte Carlo simulation (stochastic)	3 months	IRA	HIV residual risk per million donations	Additional number of new donors

HIV: human immunodeficiency virus

^a^ Stochastic parameters are described by probability distributions and deterministic parameters are fixed.

^b^ Lifetime: permanent deferral of men who have sex with men; 5 years, 1 year and 3 months: deferral of men who have sex with men for 5 years, 1 year and 3 months, respectively, after last sexual contact between men; IRA: individual risk assessment for all blood donors, with no specific deferral for men who have sex with men; no risk deferral: absence of any risk-based deferrals for all donors.

The models included a combination of the following sources of risk: (i) risk of undetected prevalent infections due to assays false-negative error rates (11 models);^18^^–^^21^^,^^23^^–^^25^^,^^27^^,^^28^^,^^30^^,^^31^ (ii) risk of recent infections in the silent window period (10 models);^19^^,^^21^^–^^23^^,^^25^^,^^26^^,^^28^^–^^31^ and (iii) risk of human error in laboratory processes (seven models).^18^^,^^19^^,^^23^^,^^24^^,^^27^^,^^28^^,^^30^ To estimate these risks, all the models applied several assumptions on the rate of men who have sex with men in the general population; the prevalence and incidence of  transfusion-transmissible infections in donors who are men who have sex with men; the false-negative rates of laboratory tests; the length of window periods; and the process error rates, based on the best available data in their context. For detailed information about the assumptions and key input variables of each model, see online repository.^13^

All the studies estimated the HIV residual risk which is the risk of an HIV-infected blood donation being received, going undetected through screening and entering the blood supply. This risk is therefore a combination of the prevalence and incidence of HIV in the donor population, the (self) deferral of donors, the sensitivity of testing assays and rates of human errors or quarantine release errors.

### Risk of bias of studies

Table 2 shows the results of the risk of bias assessment for each included model (online repository).^13^ The overall risk of bias varied and models ranged from very low to high risk of bias. The main limitation identified was the lack of validation of models, with only two studies of validated models.^27^^,^^31^ In general, the studies clearly defined their objectives, the setting and population of interest, the baseline and modelled deferral policies for men who have sex with men, and the outcomes. Three models used modelling methods which were either inappropriate or poorly described and were therefore not reproducible.^19^^,^^22^^,^^30^ Several models did not use appropriate data sources or did not justify all underlying assumptions.^18^^,^^19^^,^^22^^,^^26^^,^^30^ We identified limitations in the reporting, interpretation and discussion of results, and/or their uncertainty, in nine models.^18^^,^^19^^,^^21^^–^^24^^,^^26^^,^^30^^,^^31^ Two studies did not report a sensitivity analysis.^18^^,^^24^

**Table 2:** Risk of bias assessment of individual modelling studies on the effect of donor exclusion criteria on blood safety and volume of donations

Model, by risk of bias	Score by category						
	Definitions (0–8)	Model methods (0–4)	Model inputs (0–6)	Fitting and validation (0–4)	Results (0–4)	Conflict of interest (0–2)	Total (0–28)
**Low risk (total score of ≥ 19)**							
Davison, 2019^27^	7	4	5	2	4	1	23
Anderson, 2009^20^	7	4	6	0	4	2	23
Caffrey, 2022^31^	7	4	3	2	3	2	21
Pillonel, 2020^29^	7	4	5	0	4	1	21
Davison, 2011^21^	7	3	4	0	3	2	19
O'Brien, 2020^28^	6	4	4	0	4	1	19
**Medium risk (total score of 15–18)**							
Davison, 2013^23^	7	3	4	0	2	2	18
Germain, 2014^24^	6	3	5	0	3	1	18
Pillonel, 2012^22^	7	1	3	0	3	2	16
Yang, 2016^26^	5	3	3	0	3	2	16
Germain, 2003^18^	7	4	2	0	2	0	15
**High risk (total score of ≤ 14)**							
Aubé, 2022^30^	7	0	2	0	3	2	14
Soldan, 2003^19^	7	1	3	0	2	0	13
Ginsberg, 2016^25^	5	3	0	0	3	1	12

Note: The grading scale has 14 questions on definitions, model methods, model inputs, fitting and validation, results, and conflicts of interest. Each question was scored 0, 1 or 2 points, resulting in an overall score ranging from 0 to 28.

### Synthesis of findings

Detailed tables of findings, including the overall certainty of modelled evidence (for each outcome) are in the online repository.^13^ Nine studies modelled the HIV residual risk per million donations as outcome of the model.^18^^,^^19^^,^^21^^–^^23^^,^^27^^–^^29^^,^^31^

**Fig. 2:**
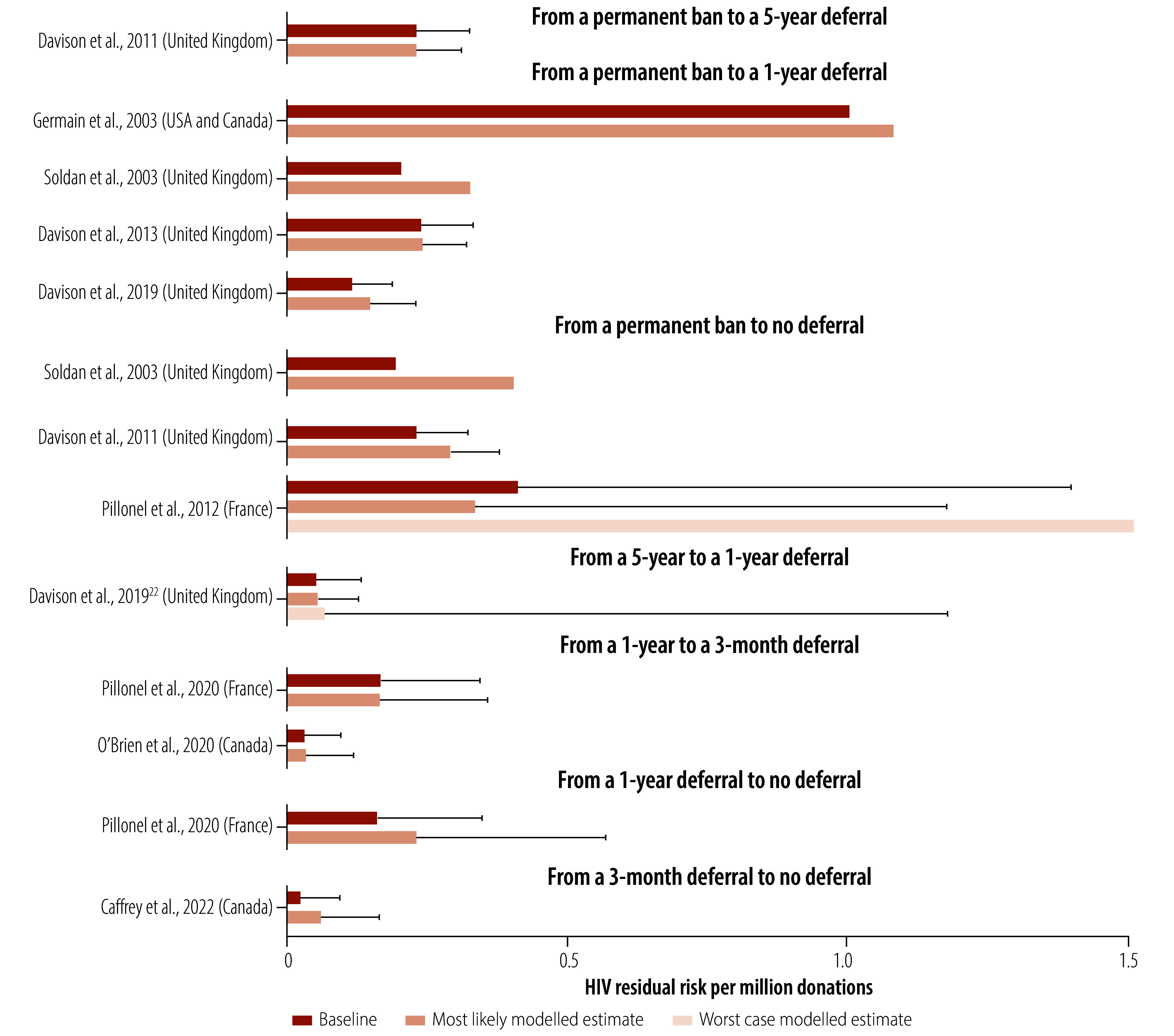
HIV residual risk in models of reduced deferral lengths on blood donations from men who have sex with men

Table 3 gives an overview of the modelled estimates of outcomes per intervention. Absolute increases in HIV residual risk were observed in all plausible scenarios, ranging from 0.004% to 300%. In absolute numbers, this translated to increases ranging from 0.001 to 0.120 additional HIV-positive blood units per million donations. For the World Health Organization European Region, estimating that 17 million donations are made yearly, this figure would translate to between 0 and 2 additional infected blood products per year.^32^ The modelled residual risk under relaxed policies on donations from men who have sex with men remained low in all plausible scenarios assessed, ranging from 0.05 to 1.10 per million donations (Fig. 2).

**Table 3:** Modelled outcomes of changes in policies on blood donation from men who have sex with men

Model	Modelled effect estimates		
	HIV residual risk [certainty of evidence]	Additional number of HIV or HBV-positive blood units detected annually [certainty of evidence]	Additional number of donors or donations [certainty of evidence]
From permanent ban on donation to a 5-year deferral	0.004% or 0.001 additional HIV-positive units per million donations [moderate]^20^	0.03% or 1 additional HIV-positive unit every 6 476 years (95% CI: 2005–16 545 years) [moderate]^20^^,^^21^ 0.004% additional HBV-positive units [moderate]^24^	Annual increase in new donors of 0.04–0.17%, equating to 4530 additional donors [low]^24^^,^^29^^,^^30^
From permanent ban to a 1-year deferral	0.004–65% or 0.001–0.12 additional HIV-positive units per million donations [low]^18^^,^^23^^,^^27^	0.1% (HIV-positive units) [low]^19^^,^^20^ 0.019% (HBV-positive units) [moderate]^24^	Annual increase in new donors of 0.88–1.3% [moderate]^18^^–^^20^^,^^23^^,^^25^^,^^27^
From permanent ban to no deferral	Effect ranging between increases of 26% to 370% (worst-case scenario), corresponding to between 0.01 and 1.1 (worst case) additional HIV positive units per million donations [very low]^19^^,^^21^^,^^22^	49 additional HIV positive blood units annually in the USA [very low]^26^	Annual increase in donors of 0.75–2.1% [very low]^19^^,^^22^^,^^25^
From a 5-year to a 1-year deferral	0–30% increase or 0.000–0.015 additional HIV-positive units per million donations [moderate]^27^	Not modelled	Not modelled
From a 1-year to a 3–4 month deferral	1–11% increase or 0.000–0.003 additional HIV-positive units per million donations [moderate]^28^^,^^29^	Not modelled	Annual increase in donors 0.24%, or 446 additional yearly donors [moderate]^28^^,^^29^
From a 1-year deferral to no deferral	43% increase or 0.07 additional HIV-positive units per million donations [moderate]	Not modelled	Not modelled
From a 3-month deferral to no deferral	300% increase in HIV residual risk per million donations, or 0.04 additional HIV-positive units per million donations [moderate]^25^	Not modelled	Annual increase in donors of 1.67% [moderate]^31^

CI: confidence interval; HBV: hepatis B virus; HIV: human immunodeficiency virus.

Note: further results are presented in Fig. 2.

HIV: human immunodeficiency virus.

Note: Error bars indicate the distribution of estimates from the Monte Carlo simulations.

Additionally, moderate certainty of the modelled evidence indicated that increases in donors from easing deferrals for men who have sex with men were relatively small (between 0.04% and 2.10% increase in total donations) for all comparisons.

### Sensitivity analysis

While only a few models conducted sensitivity analyses, 12 papers assessed uncertainty by modelling several scenarios, assuming different hypotheses about different parameters of the model. The following parameters affected the estimated outcomes: the prevalence and incidence of HIV among the blood donor population and men who have sex with men population;^20^^–^^23^^,^^27^^–^^31^ rates of donor non-compliance with deferral rules (that is, not reporting risk behaviour at donor screening);^19^^,^^20^^,^^23^ and the length of the window period.^21^^,^^27^ Testing error rates did not significantly influence the outcome estimations.^27^^,^^30^ Details of individual papers are in the online repository).^13^

## Discussion

We identified 14 papers with models estimating the HIV residual risk, and one also estimating the HBV risk, in different scenarios of eased deferral policies on blood donation by men who have sex with men. Twelve of the papers also reported the effect on the number of blood donors or donations.

The modelled HIV residual risk increased slightly with eased policies for men who have sex with men; however, differences were often (very) small and the estimated residual risk remained very low (between 0.05 and 1.10 per million donations) in all plausible scenarios. The estimated increase in blood donors or donations was also small. More inclusive policies on blood donation by men who have sex with men are therefore unlikely to have a very large effect on total blood volume available for transfusion. The interpretation of the relative increase in risk and the absolute risk may be dependent on the perspective taken. From the perspective of a blood bank that must take a cautious approach, an increase in HIV risk by 300% (the highest effect found in this review) will be intolerably high. However, this 300% increase in risk translates to an absolute increase of 0.04 additional HIV-positive blood units per million donations, which may be interpreted as a tolerable increase in risk from a societal perspective, as it is comparable to baseline risk.

The main strength of our study is the use of high-quality methods to provide the best available and most up-to-date body of modelled evidence. A limitation is that, in the absence of consensus tools on methods for the systematic reviews of modelling studies, the risk of bias was assessed using a checklist derived from the literature on modelling best practices. In this list, some of the items assessed reporting rather than quality and may not be suited for assessing risk of bias.^14^^,^^16^^,^^17^

We also identified two important limitations concerning the modelled evidence. First, the models in our review only assessed the risk of HIV transmission, and only one estimated the residual risk of HBV infection. However, HCV, syphilis and emerging diseases should also be considered when assessing blood safety. Men who have sex with men are a risk group for sexually transmitted infections, with a higher prevalence of HIV, hepatitis B and syphilis. They are also potentially at higher risk of emerging diseases that are sexually transmitted, as was seen during the first global outbreak of mpox in 2022.^33^^,^^34^ Risk-based deferral of donors is an important preventive measure for emerging diseases and for so-called escape variants of existing infectious diseases, for which no screening tests are performed or are available. Therefore, donor selection acts as the main barrier against  transfusion-transmissible infections.

The second limitation relates to the uncertainty of the modelled outcomes, resulting from several underlying assumptions. Models estimate the risk of HIV transmission in the blood supply based on assumptions such as the prevalence and incidence of HIV in men who have sex with men, the rates of donor non-compliance, and/or the length of the window period for testing. Assumptions about donor compliance might be imprecise, as it is unclear whether shortening deferral periods has an impact on donor compliance.^35^^–^^37^ It is uncertain whether deferral rules that are not seen as unduly discriminating will be better accepted and respected, and whether non-compliant donors donating under deferral rules would continue to donate after the policy change, thereby reducing overall non-compliance. As donor non-compliance was shown to affect the estimated risks in the sensitivity analyses of the models included in this review, the uncertainty around rates of donor non-compliance might affect the modelled estimates of risk. 

The use of pre-exposure prophylaxis to reduce HIV transmission might pose a risk to blood safety. Although extremely rare, breakthrough infections might go undetected due to the medication suppressing viral loads below the detection limits of screening assays.^38^ In parallel, treatment-as-prevention strategies and newer long-acting pre-exposure prophylaxis formulations are further transforming HIV epidemiology and prevention.^39^ The increasing availability of pre-exposure prophylaxis for high-risk groups has the potential to significantly reduce the prevalence and incidence of HIV, but may increase the prevalence of other sexually transmitted infections in men who have sex with men.^40^ The prevalence of infections in the donor population is a key parameter to modelling the blood safety of evolving deferrals for men who have sex with men. Thus, future models should aim to include the effects of pre-exposure prophylaxis in their underlying assumptions.

Decisions on blood donor deferral policies for men who have sex with men should be informed by both estimates from modelling studies, which investigate potential policy changes, and by observational studies, which evaluate the impact of current or past policies using real-world data. Future research on this topic should integrate both forms of evidence to provide a comprehensive understanding of blood safety risks. With the availability of high-quality, validated models, we urge blood institutions considering policy updates to assess the associated risks in their specific contexts through modelling. At the same time, they should draw on insights from observational studies, both for HIV and other  transfusion-transmissible infections. All the models we identified estimated blood safety in Canada, USA and high-income European countries, which have a specific epidemiological context, such as low HIV prevalence, and use sensitive nucleic acid tests for screening. Therefore, our results cannot be extrapolated to other contexts with different blood donor demographics, and different prevalence and incidence rates of HIV or other  transfusion-transmissible infections. As the risk of HIV in blood transfusion is related to blood donors being unknowingly HIV positive, the residual risk might be higher in countries that are struggling to reach one of the HIV elimination targets of 90% of people living with HIV being diagnosed.

As some of the models in our review were conducted several years ago and were followed by a policy change, the outcomes of the modelling can be compared with observed data. In 2016, the estimates of the six modelling studies conducted between 2003 and 2013 were compared with the observed HIV prevalence in donors after the introduction of 5-year and 1-year deferrals for men who have sex with men in Australia, Canada and the United Kingdom.^41^ All six models predicted an increase in HIV-positive male donors that was higher than actually observed.^41^ This finding may indicate the limited predictive power of modelling studies. The assumptions taken by the models about future conditions may be incorrect. The decrease in HIV incidence in men who have sex with men due to the recent availability of pre-exposure prophylaxis in high-income countries was not accounted for in the models, which may explain overestimations of risks in recent models. Our previous systematic review concluded that the certainty of evidence from observational studies was very low, in part due to limitations in study design and very low base rates.^11^ The conclusions about the absence of an increased HIV prevalence in observational studies should therefore also be interpreted with caution.^11^ Recent data from countries that shifted to individual risk assessment show higher syphilis rates among donors who are men who have sex with men.^42^^,^^43^ Our review found no models estimating syphilis risk. Continuous surveillance of trends in transfusion-transmissible infections after policy changes, as well as models including HBV, HCV and syphilis, are needed to inform future policy.

Modelling studies can incorporate the prevalence of infections in donors and the effects of subsequent protective measures, such as laboratory screenings and pathogen reduction technology for plasma and platelets, to estimate the residual risk of transmission (that is, the probability that an infected blood product would be undetected in screening assays and/or released for transfusion to a patient). However, the interpretation for clinical relevance of the residual risk is not straightforward as the administration of an infected blood product may not always lead to infection and/or disease in a patient.

In our review, only one study assessed the safety of plasma and modelled the effects of pathogen reduction technology.^30^ A recent modelling study found that pathogen reduction technology for whole blood could reduce the reliance on blood donor selection for blood safety.^44^ Since false-negative errors with nucleic acid tests are rare, the main risk of infections in blood supply are infections in the window period, where the viral load is lower than the detection limits of assays. Pathogen reduction technology could further reduce this viral load to the point that blood products would be minimally infectious. Based on the model, the authors speculated that the combination of nucleic acid tests and pathogen reduction technology complemented each other to reduce risk of infections and that donor deferrals could be relaxed.^44^ While blood banking strives for harm reduction wherever possible, the financial sustainability of interventions should always be considered.^45^^,^^46^ The cost–effectiveness of donor selection, laboratory screening and pathogen reduction technology should be analysed to inform decision-making for appropriate allocation of funds for optimized blood safety.^45^

To conclude, modelling evidence from high-income countries indicates that the residual risk for HIV transmissions by blood transfusion would increase slightly if deferral policies were eased for donations from men who have sex with men. However, the absolute residual risk of HIV transmission remains very small (between 0.05 and 1.10 per million blood donations). Additionally, the impact of easing donor deferral policies on the number of additional donations is small. Decision-making on donor policy changes for men who have sex with men requires models that estimate residual risks for transfusion-transmissible infection beyond HIV.
